# Walking as a cultural act and a profit for the landscape. A case study in the Iberian Peninsula

**DOI:** 10.1007/s10708-022-10745-x

**Published:** 2022-08-24

**Authors:** Xosé Somoza Medina, Rubén C. Lois González, Marta Somoza Medina

**Affiliations:** 1 Geography and Geology Department, University of León, León, Spain; 2 Geograhy Department, University of Santiago de Compostela, Santiago de Compostela, Spain; 3 Architecture Department, CESUGA, University of San Jorge, Zaragoza, Spain

**Keywords:** Walking, Tourism promotion, Landscape, The Camino, Ribeira Sacra

## Abstract

Walking as a means of travel, when done voluntarily, becomes a cultural act that can have a beneficial effect both for the people who carry out the routes and for the space itself that is walked on. The fact of moving at a slow speed allow us to recover a more appropriate pace to enjoy the landscape, to reconnect with nature and with the position of human in the world, while improving our health. In contemporary society, some cultural tourist routes have become successful destinations, with the continuous arrival of thousands of visitors throughout the year. Thus, the historical cultural route Way of St. James has become a globally successful cultural tourism product. Close to this destination, the Ribeira Sacra, that has been recently designated by the regional government as a Cultural Landscape, with the intention of preserving its historical legacy, may be in the future a privileged destination in Galicia for walking. The research carried out allows us to ensure that this fact, taking long walks following routes with a rich cultural content, has a positive impact on the space from two different processes that are reinforced as the routes become more popular. First, from the recovery and promotion of an alternative communication network between different places. And second, through a series of laws and regulations that protect historic trails and adjacent landscapes.

## Introduction

Walking is the best way to observe the landscape, to engage with the environment around us. When walking, our gaze can contemplate the horizon without fear of losing direction or having an accident, we can talk with other pedestrians who have similar goals and objectives creating *communitas*, in addition to improve our physical and mental health. Walking, something inherent in the human being as a bipedal animal, has become in the present a social and cultural act.

The landscape that we contemplate when walking is full of meanings. The slow speed allows us to rest our gaze and question ourselves about each substantial element. It´s unlike from what happens when we cross a territory in a vehicle, when the landscape becomes a stage of passage, a transitional area between the place of departure and the place of arrival.

Walking, as an activity that requires physical exertion, needs to be sequenced with periods of rest, stops to regain strength and then walk again. The truth is that these new contemporary ways of walking and stopping to contemplate the landscape have caused changes in the land in two directions. First, promoting the recovery of old paths or the creation of new routes that carefully search landscapes. Second, through the approval of different regulations that seek to manage those trails and landscapes that have to be preserved and enjoyed at slow speed. These two processes, the enhancement of pedestrian paths and the landscape regulation, are reinforced when the positioning of the place as a tourist destination generates the growing arrival of visitors, and especially when its qualities are distinguished by a prestigious international nomination, such as being a World Heritage Site or a European Cultural Itinerary.

Within this framework, this paper study the Ribeira Sacra, a region in Galicia, in the northwest of the Iberian Peninsula, in which the physical and cultural conditions of the territory have motivated its designation by the regional government as a cultural landscape, what represents an administrative regulation of its preservation and management. Local agents have been trying to promote their tourism development for a long time, following the Camino de Santiago as a model, which year after year attracts hundreds of thousands of visitors to Galicia.

## State of the art. Walking as a cultural act

Contemporary philosophy has highlighted the role of the flâneur, that person who walks, who goes through the city, contemplating, making the walk an authentic life experience and a way to discover the world (Benjamin, 2012; Harvey, 2003). In a parallel theoretical development, British Sociology and Geography insist on the "tourist's gaze", on the sense of place and on the new culture of walking, of trekking along small paths and trails, from where the environment is contemplated (Creswell, 2004; Creswell & Merriman, 2011; Urry & Larsen, 2011). Undoubtedly, the contemporary human being needs to move, as a metaphor of life. We need to discover and feel that we are looking at the landscape, at the horizon, to find ourselves again (Coleman and Eade, 2004).

On the one hand, walking means to recover the rhythm of life of our ancestors (Lois, Castro and Lopez, 2015). It involves moving at a speed not mediated by fast machines. On the other hand, derived from the previous one, it means breaking with the daily urban rhythm of life, scheduled, frenetic, which absorbs us most of the year. This is the reason because of what walking is associated with rest, tourism, or breaking-off with the usual rules of life. In addition, walking through historical and well-preserved itineraries makes possible to contemplate the landscape calmly, to enjoy nature and the heritage of the past (Stoddard & Morinis, 1997; Lorimer, 2011). All this is part of a pleasurable and satisfying experience that is increasingly in demand.

In all the approaches to the issue, the concept of slow mobility is raised as the way to reach the place at 5 or 15 km per hour, whether we move on foot or by bicycle. This calm movement allows us to soak up the immediate environment, smell, enjoy the thermal sensation and appreciate the fields on the scale of a plot of land or a small forest. It places the body and its relationship with the environment at the centre (Urry, 2000; Creswell & Merriman, 2011). In addition, hiking and walking tours are perceived as healthy, another attribute associated with travel. The walker prepares himself with specific clothing to face the route and exercises his endurance capacity the days before. The whole experience involves a mixture of preparation, personal care and effort interpreted as a physically and mentally healthy act (Hall et al., 2018; Pileri and Moscarelli, 2020).

Walking modifies our perception of the territory, usually mediated by motor vehicles that make us contemplate the environment in a hurry, the landscape as a rapid sequence of scenes. This raises a very characteristic cultural divergence of the contemporary period. The Hegelian idea of progress led us to move continuously and quickly. Modern societies, so well analysed by the sociologists of the Chicago School, characterised the urban way of life by the continuous, rapid and often pattern displacement of the city’s inhabitants (Urry, 2006; Wirth, 1937). On the other hand, thinkers closer to our times, from W. Benjamin to current narrators, including the French philosophers of the 1960s and 1970s, have extolled those who walk slowly without a clear objective through the city, the so-called flaneurs,, converted into an icon culture of the present (Benjamin, 2012; Harvey, 2003; Simmel, 1950). If we leave fully urbanised contexts, the walker, the person who goes trekking or takes a calm itinerary through a space of heritage and landscape value appears before us as an essential reference.

Following a route or itinerary also involves crossing the landscape in between, enjoying the whole visual and mental experience of the countryside understood as nature. The landscape is enhanced by its chromaticity (and here the green vegetation brings many advantages) and by its abundance of visual elements (trees, cultivation areas, rivers and streams, houses, etc.) (Besse, 2018; Roger, 1997). For an urbanite with a programmed and overloaded schedule, it means getting rid of all the rhythm of daily life and stress. Hence, paths of all kinds are considered to trigger the feeling of liminality, of rupture between one phase of life and the next (Turner, 1969). If the route is prolonged, the *communitas*, camaraderie and sense of brotherhood among those who are on the route, arises as a second attribute. Undoubtedly, the landscape helps to generate the main attraction of the walk, the possibility of an interior trip, the time to rediscover oneself (Turner, 1969; Coleman and Eade, 2004).

As has already been mentioned, the simple act of walking has many different meanings. Urbanites, whether they are workers or students, walk and move from their homes to their workplaces on a regular basis every day. In the beginning, climatic, sun and beach tourism preferred the speed and efficiency of medium and long distance travel to reach a destination, where they could enjoy the heat, light and fun without moving too much (Urry & Larsen, 2011). But social preferences have changed profoundly. More or less intense exercise occupies our leisure and rest time (now more mental). The recovery of the value of the body in Western culture not only does us good when walking, but also stimulates the value of our senses (Lacan, 1977). We observe and enjoy the landscape visually, aurally or olfactorily. We discover in a similar way to the Impressionists how the tones and colors of objects change according to the time of day and the season of the year. All this reinforces the value of long walks, of walkability, always referred to an itinerary of cultural significance (Pileri and Moscarelli, 2020).

In this brief theoretical reflection, the thesis that remains is the contemporary recovery of landscape itineraries endowed with historical foundation. Walks, calm contemplation of the environment and sustained exercise are values appreciated by today's mostly urban populations (Creswell & Merriman, 2011). This new sensibility became evident with the recovery of the Camino de Santiago and later with the emergence of the denomination of European Cultural Routes (Edgerton, 1975; Lois González, 2013). The UNESCO World Heritage Declarations reinforce this symbolism, endowing the paths intended for walking or cycling with a brand of international prestige, which is often sought. On a more concrete level, they encourage careful interventions on the landscape, and make the natural and cultural environments become targets of intense care. Whole spaces are prepared for contemplation and enjoyment due to protective interventions that always make those innovative and more sustainable forms of tourism to be cost-effective.

## Methodology

The relationship between the space and the visitor can be different depending on the person's motivation for the journey. Thus we can distinguish among traveler, tourist and pilgrim. Anyone who makes a journey can be defined as a traveler, regardless of their motivation and the time at which they make it, but among all of them, those who make the journey as a recreational activity in their leisure time are considered to be tourists, while pilgrims are those whose main motivation is religious and who make their journey to a pilgrimage centre (Cohen, 1992). Unlike in the past, the current distinction between tourists and pilgrims is not so sharp (Eade, 1992). From the tourist-pilgrim divide that anthropologists established in the 1980s (Adler, 1985; Turnbull, 1981; Turner & Turner, 1978), we move to the tourist-pilgrim nexus (Ackerman, 2019), to the understanding that a religious or even cultural tourist can make pilgrimages and that a pilgrim can behave like a tourist. A traveler may switch from tourist to pilgrim and vice versa in the same trip without the individual being aware of the change (Smith, 1992). From an individual point of view we could say that the pilgrim's journey is fundamentally an interior journey, the person who makes the journey with a religious motivation looks within herself/himself in a liminal process and the feeling of belonging to a community formed by the religious monuments and other pilgrims is very strong. Whereas the tourist with a strictly recreational motivation looks mainly outwards with the aim of being surprised by the beauty of what can be seen in that gaze. Precisely, the combination of both views is part of the success of tourist phenomena based on pilgrimage routes such as the Camino de Santiago.

But also, as stated in the title of the article, the act of walking becomes a cultural act that can be structured in different interpretive schemes. The cultural experience of walking through historical landscapes induce three different levels of knowledge, depending on the wanderer’s involvement with the observed environment. So that, the first level of understanding refers to the learning that results from the act of encountering what is there in sight. The tourist accumulates and apprehends information that has been previously selected and prepared to be seen. The second level of knowledge is acquired through the discovery of what is veiled. The traveller reveals the substance that identifies the place, and comprehends the meaning of the unexpected. The third level of knowing leads to being aware of the causes and effects of what is seen, beyond the spatial concreteness and the present moment. The walker relates what is contemplated with the economic and social repercussion of the layers that overlap the land, developing critical thinking. This third level of understanding, the possibility of transcending through the perception of the landscape, is directly related to the combination of the two gazes that we explained before, the exterior one that analyses the landscape and the interior one that allows us to develop abstract concepts while we contemplate the exterior landscape that frames us as we walk.

The objective of this article is to analyze the contemporary attributes of the act of walking on a cultural tourist route and the positive effects that this activity can generate on the territory. To this end, the promotional and tourist success of the Camino de Santiago is analyzed first as a model, and then the Ribeira Sacra (The Sacred Riverside), is investigated as a case study. The Ribeira Sacra is a region in the northwest of the Iberian Peninsula, between the provinces of Ourense and Lugo. The research question is whether the Ribeira Sacra is an appropriate place for the promotion of a route of these characteristics in relation to the existing cultural landscape and the regulations in force for its protection and management.

The methodology used to answer the research question and fulfill the proposed objective is based on the analysis of the environmental legislation of landscape protection applied in each territory and on the repercussion that the tourist activity presents in the socioeconomic reactivation of these countries.

In this context, the article begins with a review of the theoretical contributions that have been developed around walking studies by the social sciences. Then, it is exposed how these theoretical ideas have been embodied in the promotion of the Way of Saint James (Camino de Santiago), turning it into a contemporary cultural product. Finally, it is presented the Ribeira Sacra study case, after what some conclusions are exposed.

## Model of success. Walking in the Camino de Santiago (Way of Saint James). A contemporary cultural route from the Middle Ages

The Caminos de Santiago are a series of pilgrimage routes of medieval origin that lead to the place where, according to Christian tradition, the tomb of the apostle St. James is located. Around 830, the hermit Paio revealed to the bishop of Iria Flavia that at night he saw lights pointing to a specific place. In that place, the discovery ("inventio") of the Marble Ark with the remains of the apostle took place. When King Alfonso II was warned, he went there, making from Oviedo the first pilgrimage to Santiago in history, to verify the discovery, ordering the construction of a temple to honour the holy relics. Two centuries later, in 1095, under the influence of the first archbishop of Santiago, Diego Gelmirez, Pope Urban II declared the remains to be true, confirming the title of Western Apostolic See. Archbishop Diego Gelmirez is the key figure at this beginning of the Camino, as he was able to establish relationships of influence and opportunity in several important places of the time (Rome, Cluny, Porto, León), to conclude the works of the new Cathedral, to plan the new city and to promote internationally the pilgrimage to Santiago (López Alsina, 1999). Since then, and for centuries, the pilgrimage routes have become axes of cultural and socioeconomic development, with religious and military orders founding churches, monasteries and castles along them.

The reinvention of the Camino de Santiago is much more recent (Santos Solla, 1999; Lois González, 2013). It was restarted during the Franco dictatorship, when the government promoted the restoration of numerous religious buildings along the Camino to vindicate the role of the Catholic and conservative memory of Spain at the time (Castro Fernández, 2010). It continued with the incorporation of Spain in 1985 to the European Economic Community, when the Spanish government adduced the historical pro-European character of the Camino. Two years later, the Council of Europe named the Camino de Santiago as the first European cultural itinerary. Afterward, the autonomous communities crossed by the Jacobean routes began to promote these axes as tourist products. Since the fifteenth century, the Compostela Holy Year has been celebrated every time the feast of St. James, July 25th, falls on a Sunday. For the Galician autonomous community, the 1993 Holy Year was the moment chosen to exploit the Way of St. James for tourism, culminating a whole series of processes that had begun earlier. Such as the construction and rehabilitation of pilgrim hostels, the delimitation and conditioning of the routes, the international promotion of the Camino together with the Associations of Friends of the Camino, the urban rehabilitation of the city of Santiago or the hiring of a series of events and cultural ambassadors who spread around the world the benefits of the Camino. Finally, in December 1993, the UNESCO included the Camino (The French Way) in the World Heritage list (Santos and Lois González, 2011).

**Fig. 1:**
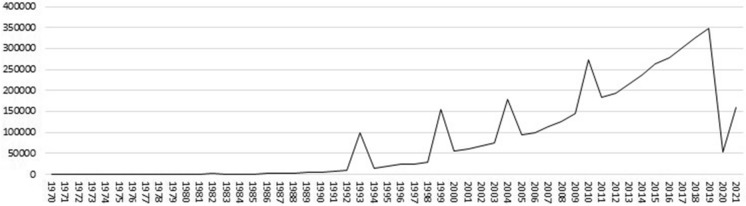
Pilgrims to Santiago de Compostela 1970–2021. (Pilgrims Office, 1970–2021)

The fact is that the few dozens of pilgrims who came to Compostela in the mid-twentieth century increased up to 347,578 pilgrims in 2019 (Fig. 1). The main route was The French Way, according to statistics published by the Pilgrim's Office (Oficina del Peregrino, 2021) with 189.937 pilgrims (54,6%) but the second and third in order of importance were routes originating in Portugal: the Portuguese Way (72,357; 20.8%) and the Portuguese Coastal Way (22,929; 6.4%). They arrived from 189 different countries. A part of them, with religious professions other than Christianity, made pilgrimage to Santiago for cultural, tourist, or personal reasons. Nowadays, the Christian medieval route has become a successful contemporary and global cultural tourism product (Lois González et al., 2015).

The current success of the Camino is not a circumstantial fact, nor is it the product of a fortunate political project. It is much more. On the Caminos de Santiago there are the confluence of a series of strengths and opportunities, of positive elements of an internal and external condition, which generate a process of continued growth that must be planned and managed to ensure its sustainability.

Although the reinvention of the Camino is a contemporary event, which creates a cultural itinerary aimed at people of the present, far removed from the motivations of the medieval pilgrim, a series of elements give uniqueness and enormous wealth to this route, which differentiate it from the rest and serve as a model for new areas in the process of qualification such as the Ribeira Sacra. Among them: (a) the material recovery of the paths, prepared in such a way that the walker's experience is similar to that of centuries ago. The land is conditioned, trees are planted along the edges, and stones or cobblestones are added in certain places (Lois González, 2013). In the Ribeira Sacra, all trekking or cycle tourism routes seek to generate the same experience; (b) a comprehensive rehabilitation of the road space towards Santiago has been carried out for decades (Castro Fernández, 2010; Somoza Medina & Lois-González, 2018). In the Ribeira Sacra, the intervention in Romanesque churches and monasteries, which have even been converted into luxury hotels, has been a priority; (c) the public image of the Camino continually repeats the value of history, tradition and spiritual rites associated with an intense physical practice and experience that is maintained over many days (Lois González, Castro & Lopez, 2015). In the Ribeira Sacra, the vindication of the heroic viticulture that began with the Romans, the austere monastic way of life and the effort to build the cultivation terraces on the slopes of the river, remains in the memory of the visitor, particularly in those who makes an intense effort when walking or pushing a bicycle on rural trails. In June 2022, 31 municipalities from the provinces of León, Ourense and Lugo have come together to promote a route of more than 200 km that runs along the entire Sil River, culminating the journey, the last 50 km, in the Ribeira Sacra.

To walk the Camino de Santiago is to resort to slow speed, on foot, to reconnect with oneself and with the land we tread, while enjoying a cultural landscape with centuries of history. To travel a Jacobean route is to have fun with a physical effort with immediate social and cultural reward. Doing the Way involves to enjoy the first European cultural itinerary and to share experiences with other pilgrims, while staying fit.

**Fig. 2:**
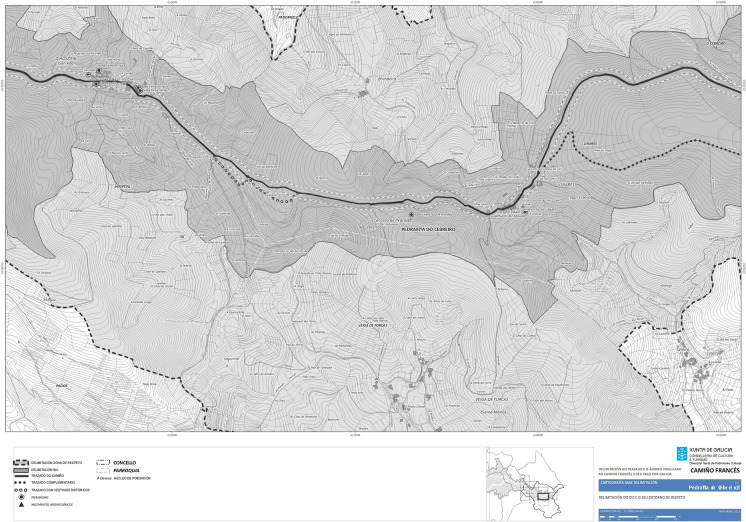
Demarcation map of the Camino in Pedrafita do Cebreiro. Source: Xunta de Galicia

But in order to fulfill these contemporary motivations it has been previously necessary to improve the walkability of the routes (Somoza Medina and Lois Gonzákez, 2018). With this objective, the different historical routes have been officially demarcated, with detailed planimetry and bounding areas of protection on both sides of the route (Fig. 2). In a later process, these paths have been developed, improving the sections, crossings, or pavings, conditioning rest areas and creating new service points.

On the other hand, the regions have legislated specific protection measures for the Camino, as in Galicia the Law 3/1996 on the protection of the Caminos of Santiago, or the decrees approved in Asturias, Castilla y León, Navarra or Euskadi for the delimitation and protection of the Camino in their regions, or the 51/2019 decree for valorization and promotion of the Caminho in Portugal. The Spanish documents lay down the routes and the lateral protection zones on both sides of the walking axis, with a minimum width of 3 m, the environmental protection zones, with a width of 30 m also on both sides and the buffer zone. In those lateral protection zones uses incompatible with the Camino are prohibited and any action is subject to prior administrative approval (Somoza Medina & Lois-González, 2017). The Portuguese decree establishes the requirements to certify the itineraries that request it with the title of Camino de Santiago. The declaration proposal must include for each section of the trail the specific conditions of safety, walkability, support equipment and synaletic.

As for the landscape, beyond those 30 m and the buffer zone, Portugal and some autonomous communities in Spain like Catalonia or Euskadi are also legislating landscape protection measures around the Caminos. For instance, Galicia successively approved the Landscape Law of Galicia in 2008 and in 2020 the Landscape Guidelines. This guidelines make specific reference to the protection of all those elements that constitute the cultural landscape of the Camino de Santiago and the minimization of the affections to the structural lines of the landscape linked to the Way: linear elements (traditional closures, hedges, woodland, etc.), adjacent road system, watercourses, land pattern or crop system (Xunta de Galicia, 2020).

Public administrations have also continued to finance the rehabilitation and restoration of the public heritage built in the area and to grant aid to the population to rehabilitate private buildings and maintain the socio-productive fabric along the routes. The surroundings of the Camino de Santiago are privileged enclaves in this sense, because in peripheral rural areas disconnected from the main development axes, there are public and private investments that converge and generate a differentiated situation with respect to neighbouring territories. This circumstance leads other municipalities to try to recover or reinvent new roads to Santiago that run through their localities.

**Fig. 3:**
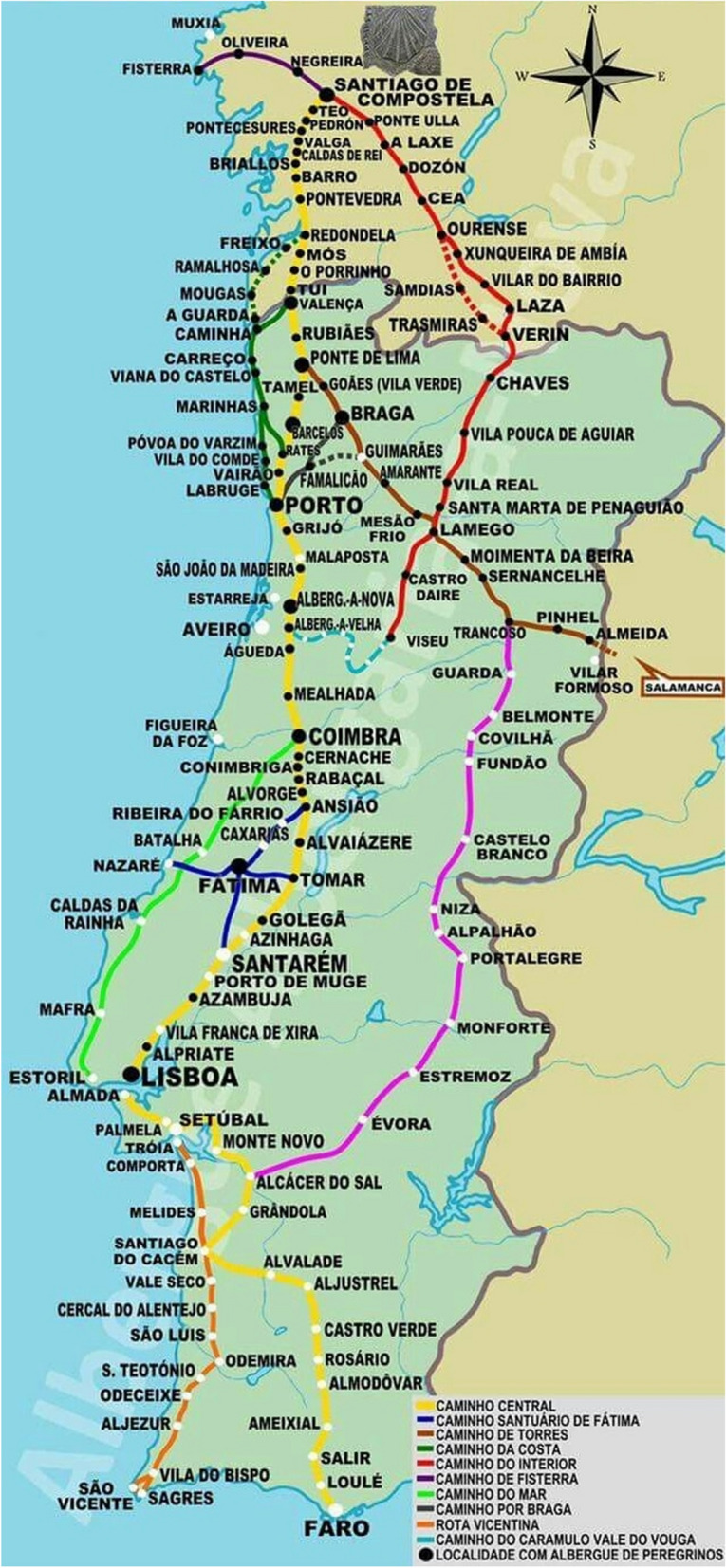
Caminhos de Santiago in Portugal. Source: Albergaria A Nova

**Fig. 4:**
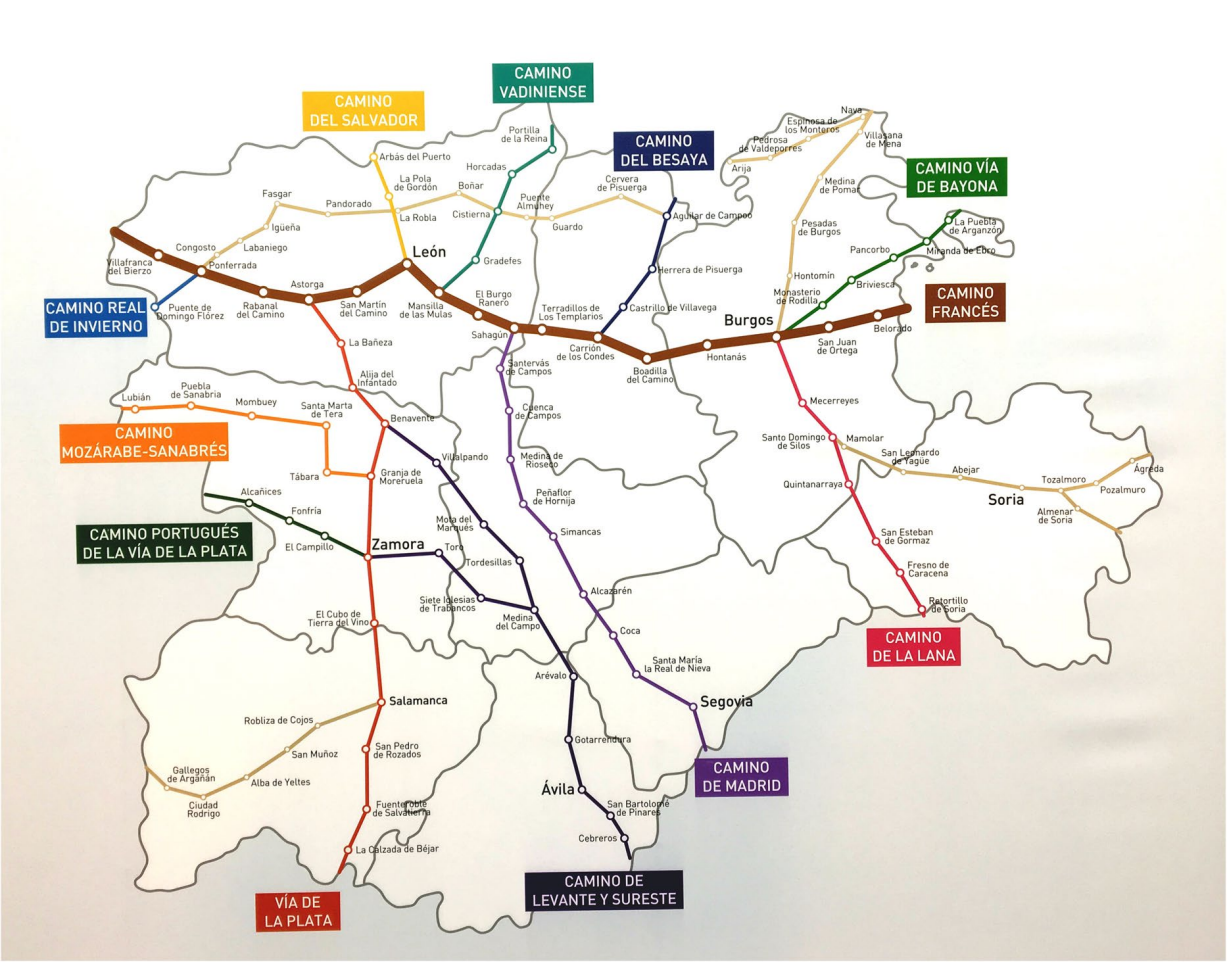
Caminos de Santiago in Castile and León region. Source: Junta de Castilla y León

This phenomenon, the diversification of the Jacobean routes, is also a strategy adopted by the different governments (Somoza Medina & Lois-González, 2017) and embodied in planning documents as can be seen in the cases of Portugal and Castilla y León (Figs. 3 and 4). The objective is not to saturate the Camino and die of success, by extending the Jacobean phenomenon and promoting all the Ways to Santiago and within them the main routes and their historical and complementary or functional variants, which may deviate slightly from the original route to visit other places or avoid high traffic roads (Fig. 2).

In summary, the Caminos de Santiago treasure all the best features of the current tourist and cultural routes, on which contemporary society will continue to walk in the coming years, first to get lost and thus be able to find themselves.

## Case study. Walking in the Ribeira Sacra? Success possibilities of new cultural routes

Many websites offer touristic routes to walk through the Sacred Riverside, promising the most genuine experiences. Expressions such as “spectacular landscape”, “wonders of nature”, “more than attractive hiking trails” or “its purest rural essence” invite people to discover a region that it is distinguished from other places by certain identity values of its own.

Those identity values have led to the declaration by the Autonomous Community of Galicia in 2018 of a large area as the first Cultural Landscape, conceding protection to the built elements and the immaterial assets that manifest the relationship over the centuries between the community and their territory. This legislation imposes administrative authorization prior to any action on the declared assets, the prohibition of incompatible uses with those of a cultural landscape, the duty of conservation to the owners and the obligation to allow public visits. The Ribeira Sacra is the oldest site in the Spanish Tentative List of UNESCO World Heritage Sites, since 1996. In 2019, UNESCO introduced part of this place in the Global Geoparks list (Courel Mountains) and has declared in 2021 the region as part of the Biosphere Reserve "Ribeira Sacra, Serras de Oribio e Courel", with the objective to balance the sustainable development with the preservation of natural and geological resources, biodiversity and cultural heritage.

**Fig. 5:**
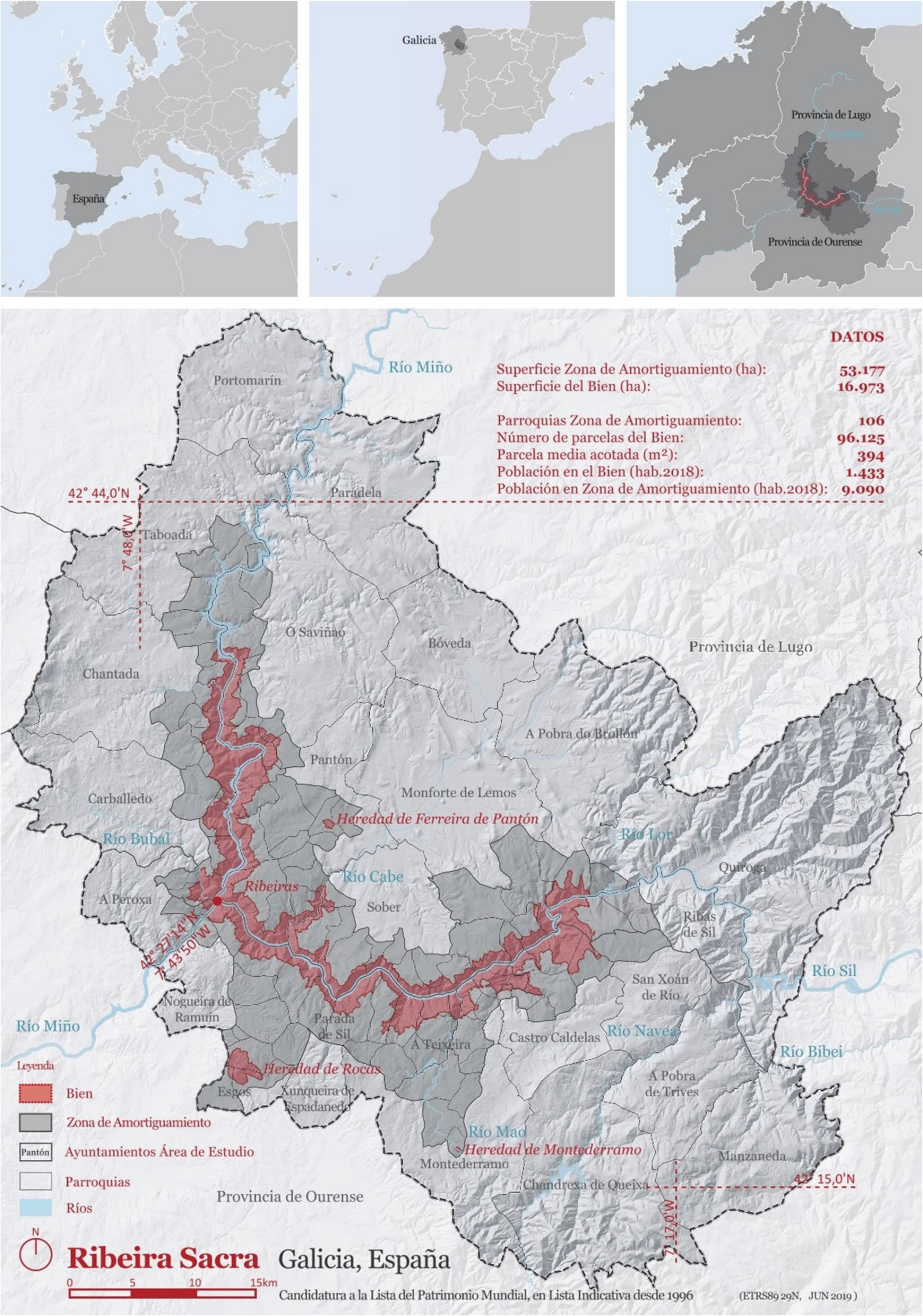
Sacred Riverside map. Source: Xunta de Galicia

The Ribeira Sacra, located in the northwest of Spain, shows a cultural landscape with a geomorphological substratum of river valleys, strongly anthropized both in the past and in the present (Pérez Alberti, 2019). The peaks reach 1.200 m., with steep slopes and small plains interspersed at different levels. Over the centuries, the communities that have inhabited this region have shaped it, generating a historical territory in which strata are superimposed, showing the different stages of relationship between human and nature, as a *palimpsesto* (Corboz, 2020). This landscape accumulate rests of fortified ancient settlements in high positions, Roman infrastructures for gold mining, religious communities with foundations from the sixth century onwards, paths from hundred of years like the Winter Route of St James, terraces formed on the riverbanks for vine cultivation and the more recent infrastructures for energy production by using the power of water and wind (Fig. 5).

The main promoter body in the area is the Ribeira Sacra Tourism Consortium, which was created in 2005 with the aim of planning, managing and promoting the development of the area, implementing the Ribeira Sacra Tourism Promotion Plan. It is currently composed of 21 municipalities, the provincial councils of Lugo and Ourense, the group of winegrowers and wine entrepreneurs of the Ribeira Sacra Designation of Origin and the Association of accommodation and active tourism companies Ribeira Sacra Rural. This Consortium implemented between 2006 and 2008 a Tourism Promotion Plan endowed with 3 million euros that defined this country as a special destination, not massive, with an innovative product based on heritage formed by the sum of landscape, monasteries, rivers and wine, and with added value for a sense of place of spirituality. The confluence of public and private investments has generated in this rural area since then a considerable hospitality infrastructure (Table 1), and a network of touristic services that offer the visitor a varied supply of complementary activities (Table 2).

**Table 1:** Hospitality offer in the Sacred Riverside

	2003	2008	2013	2018	2022
Four-star hotels	1	3	3	4	5
Other hotels	19	21	24	25	26
Guesthouses	–	–	40	41	52
Pilgrims hostels	–	–	17	33	37
Rural Tourism houses	39	42	44	46	50
Houses in hospitality platforms	0	0	4	50	290

Source: www.ige.es

**Table 2:** Tourist services in the Sacred Riverside in 2022

Restaurants	50
Touristic cellars	35
Museums	21
Viewpoints over the valleys	49
Active hiking tourism companies	14
Traditional ceramic factories	5

Source: Ribeira Sacra Tourism Consortium

**Fig. 6:**
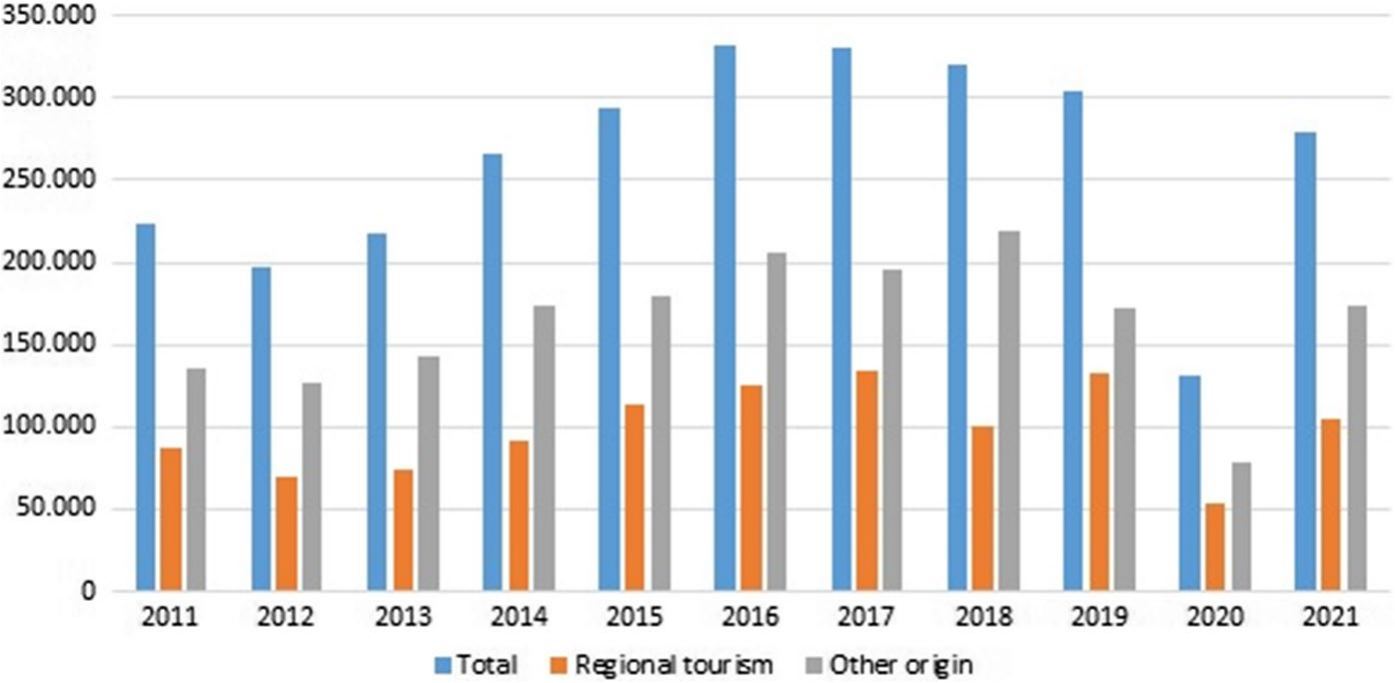
Number of touristic nights in Ribeira Sacra 2011–2021. Source: www.ige.es

The Promotion Plan marked the beginning of the tourist transformation of the Ribeira Sacra. The number of visitors have increased continuously since 2006, until the crisis caused by the COVID-19 pandemic. Figure 6 shows the evolution of the number of tourist overnights among 2011 and 2020, highlighting the increase of tourists from outside Galicia.

In this territory, the link among landscape, tourism and wine has been highlighted since the Promotion Plan and the evolution of the rates of the production and commercialisation of the wine of the Ribeira Sacra clearly shows the development experienced in recent years. Table 3 underlines the growth of production, although the number of winegrowers has been reduced, and the importance of foreign sales, with the main export markets being the United States (31% of the total), Norway (10.3%) and the United Kingdom (10.2%). Many visitors know first the wine and then the heroic viticulture of this tourist destination, while other hikers visit the wineries before knowing the excellence of their wines.

**Table 3:** Main indicators of the Denomination of origin Ribeira Sacra

	2003	2008	2013	2018	2021
Kilos	3.311.618	3.975.314	4.735.899	6.172.222	6.541.212
Hectoliters	22.105	26.566	31.033	38.943	43.076
Hectares	1.200	1.228	1.250	1.238	1.276
Winegrowers	2.807	2.836	2.817	2.376	2.291
Cellars	94	98	90	94	98
Exports (Hls)	–	26,55	137,46	2.136,93	1.901,65

Source: DO Ribeira Sacra

In December 2020 the Tourism Sustainability Plan was approved with a budget of 2 million euros contributed by the national administration (42.5%), regional government (42.5%) and the Consortium (15%). The objective of this Plan is to launch a series of actions, in the frame of the candidacy of Ribeira Sacra to World Heritage, to ensure environmental, economic and social sustainability in the process of consolidation of Ribeira Sacra as a tourist destination. The plan is articulated in five pillars: sustainable mobility; cultural landscape; competitive transformation; product, marketing and tourism intelligence; and participatory governance.

**Fig. 7:**
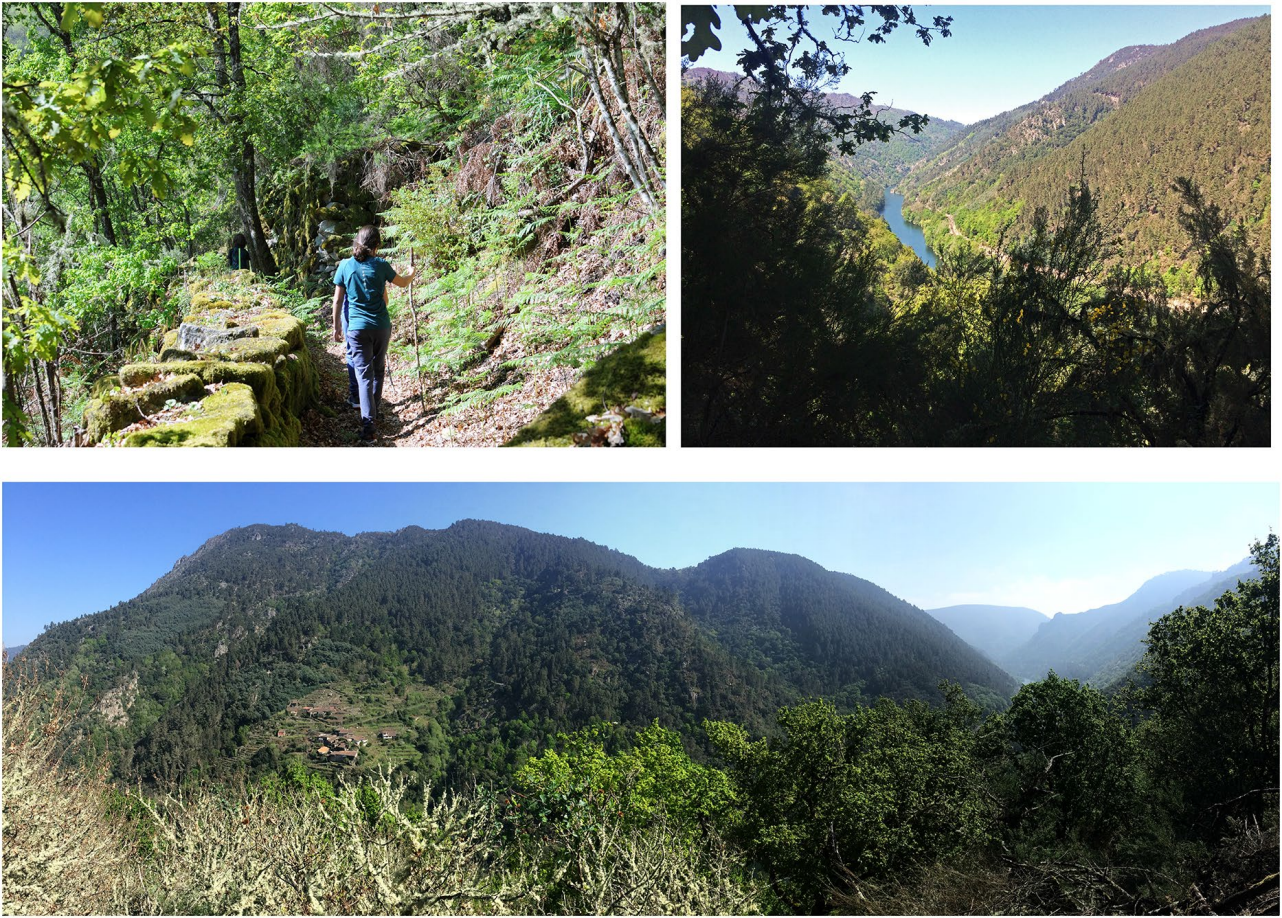
Landscapes of the Sacred Riverside from different trails. Source: Marta Somoza

As a part of the development of these goals, the consortium has created a large and diverse offer of walking routes, which, while relying on the traditional path network, incorporate elements that improve or complete their accessibility and walkability (Fig. 7).

The morphological features that the walker observes include embedded valleys with steep slopes, presence of water in rivers and streams, alternation of wooded masses (birch, oak, chestnut), vineyard terraces and areas of scrubland, winding lanes combined with linear stretches of tracks and footbridges, and granite textures in paving, side walls, and constructions. The perceptive attributes of the pahways combine landscapes with limits given by the sequence of planes at the average distance and landscapes without limits (Barba, 1988), alternating the limited visibility of the enclosure with the panoramic view of the summits of the canyon. The views from the paths are characterized by diversity, variability and surprise. The amount of elements observed from a close approach allows the small, the particular and the heterogeneous to come to the surface; thus, the multiplicity of forms of micro-topography, vegetation, watery beds and manufactured works compound singulars scenes where texture, colour, dynamicity and light append even more quantity of diverseness. In addition, the low walk also introduces the time required to identify the variation of certain pieces in a sequence; the capability of dynamical systems and fractal geometries to change from a previous registered state or order induce the game of finding differences, developing an active attitude in bystanders despite the unawareness of the action. On the other hand, the feature of surprise connects with the idea of what is not expected and consequently, with the introduction of an alteration of a given rhythm, introducing landmarks in the temporal and spatial becoming of the ramble.

The re-elaborated path network stand these qualities out by incorporating the following points:

1. Circular trajectory, which allows: a high degree of sensitive diversity, avoiding return along the same place; the apprehension of the variation of contiguous items along the progressive advance; and the unforeseen apertures in the frond due to gradient changes in the paths' ups and downs.
2. Dotted missions that propose a list of assorted natural and architectural elements, whose collection produces a program to be followed; that display alterations inside specific tipologies (geologic fault, bridge, hermitage, …); and that startle because of the discovery of precious treasures.
3. Viewpoints, where the enlarged field of vision allows the recognition of the place in its amplitude, seen as a sum of countless components, with variations in the land cover pattern and the revelation of the openness. The lookout also makes possible the perpetuation of the moment through selfies (Dinhopl and Gretzel, 2016; d’Eramo, 2020).
4. Spaces for the pause, the encounter and the event, defined between the introspection of the reflective sight of the surroundings and the extraversion of the dialoguing attitude with neighbouring beings; between the fluctuation in human density and social relations through time, an between the commotion given by the welcome of the rest and the joy of the shared happening.

## Conclusions

Walking, in today's fast-paced and stressful world, has become a cultural event, and a healthy, enriching and pleasant tourist activity. International organizations, such as UNESCO or the Council of Europe, aware of the importance of tourism and culture associated with specific journeys, have promoted their conservation and furtherance by including them in lists of world recognition. The inclusion of a trail in any of these lists implies the achievement of an objective sought by local and regional administrations, which assume that this designation will facilitate its conservation and socioeconomic development.

In the Iberian Peninsula, the Caminos de Santiago is the route par excellence that treasures all these contemporary virtues. Based on its success, different local and regional governments have implemented other itineraries with the aim of attracting resources and tourism to their territories.

This is the case of the Ribeira Sacra, a region marked by the historical adaptation of human beings to a complex geographical environment. In the Ribeira Sacra, the coordinated work of public and private agents over the years has led to its candidacy to be part of the UNESCO World Heritage List. For this, one of the most definitive step has been the designation of this area as a Cultural Landscape, a legal definition with measures for the protection and conservation of landscape and the promotion of tourism, as well as a precise description of their tangible and intangible assets. The regulations generated by this legal designation pretend to preserve this territory from future impacts on its structural elements.

The tourist promotion of an extensive area such as the Ribeira Sacra is carried out through different proposals which include the renewal and completion of a network for slow travel modes like bike, boat or foot. The walkability underline the sensitive perception, the active attitude and the sense of progress through movement. The individual space created by walking melt with the geographical space, so that the general overview that summarizes the economic and social organization of a certain territory is enriched with the nuances given by the particular, the small and the heterogeneous. The landscape is therefore not only observed but interiorized, experienced and overwrite by the awareness of its presence and the whole of meanings beyond its materiality.

Beyond the values directly associated to the act of walking through cultural landscapes, the improvement of the walkability infrastructure can become a powerful tool to manage the rural territory of Ribeira Sacra. In light of all the above, there are four interwoven ideas to be explored in order to further the sustainable development of the region: the structuring capacity of the network of paths understood as a system, the enhancement of the morphological features, the achievement of ecological balance and the reinforcing of the identity and the belonging feeling.

- Guided mobility can be seen as a nervous system of channels and nodes where crossings draw a mottled map of use, and whose planning should balance accessibility, connectivity and intermodality (Parcerisa and Rubert, 2002). The idea to implement a network that would bring together all the walk trails in a system of lines that intersect at nodes would improve the connectivity between tourist destinations and also between hamlets; it would favour the arrival to sites that have lost easy accesses, and it would include exchange of different means of transport.
- Simultaneously, the network system of paths would create centralities in public spaces located in certain crossings of the net, with specific functions and spatial dispositions. The urban project to fit them should lead to the exaltation of the site by taking in account its morphological features. These characteristics are similar to some of those defined by Professor M. de Solà Morales (2020) when speaking about Mediterranean metropolises: the value of the topographical enclave, the small grain of plots and the open communication generated by a profuse network of roads.
- Meanwhile, natural systems are important to provide air, water, food, biodiversity and leisure for a large population (Forman, 2014). The consideration of the path’s network as a land structure could help to achieve certain landscape ecology balance favouring compensated interactions between people and nature. The mere visibility of the territory from the paths can be used as a local monitoring mode to prevent situations that resulted in the deterioration or destruction of ecological values.
- From a territorial scale, Sieverts (2015), in order to explain the inter-city territories of large urban regions, reviews the concepts that defined the good city, such as urbanity, centrality, social density and the mix of uses. The urban planner considers that the legibility and comprehension of intermediate landscapes would allow to experience the region as the space that shapes the everyday life. Therefore, the greater frequentation of the network by the inhabitants of the region and the consequent production of personal and communal experiences in different places would strengthen the recognition of the characteristics of the territory and the feeling of belonging to the place.

For the sustainable future of this territory, slow travel modes must be enhanced. Several short walking routes have been designed, to be done in a maximum of 6 h of walking. It would be advisable to offer longer routes, which require the use of different modes of transport through trails, rivers and roads, perhaps two routes on each side of the Ribeira Sacra which, over the course of several days, would allow to achieve the three levels of knowledge that were mentioned. Maybe something similar to the Stevenson Route in France (Chemin de Stenvenson, GR 70). Longer routes that would combine the exaltation of the landscape and cultural heritage, the creation of comunitas with other walkers and the personal satisfaction of rewarded physical exercise.

Therefore, the enhacement of the walkability infrastructure, which illuminates certain routes over others, can become a valuable tool for the territorial organization. This kind of guided mobility system can improve the connectivity and accessibility of the hamlets, can allows intermodality and can produce small centralities in the public spaces of certain intersections, favouring the concentration of social relations.
